# Supplementary material for: Modeling glioblastoma heterogeneity as a dynamic network of cell states
Source: Mol Syst Biol. 2021 Sep 16;17(9):e10105. doi: 10.15252/msb.202010105 (PMC8444284; doi:10.15252/msb.202010105)
Supplement: Supplementary file 6 — Source Data for Figure 5 [file MSB-17-e10105-s004.zip › Figure5A_sourcedata/GSEA_3071/nosigdb_stateIV.GseaPreranked.1622795164626/NB_KI_CLUSTER2.html]

Details for gene set NB\_KI\_CLUSTER2[GSEA]

|  || Dataset | state53071 |
| Phenotype | NoPhenotypeAvailable |
| Upregulated in class | na\_pos |
| GeneSet | NB\_KI\_CLUSTER2 |
| Enrichment Score (ES) | 0.47949338 |
| Normalized Enrichment Score (NES) | 2.8558521 |
| Nominal p-value | 0.0 |
| FDR q-value | 0.0 |
| FWER p-Value | 0.0 |
Table: GSEA Results Summary

  

Fig 1: Enrichment plot: NB\_KI\_CLUSTER2      
 Profile of the Running ES Score & Positions of GeneSet Members on the Rank Ordered List

  

| PROBE | GENE SYMBOL | GENE\_TITLE | RANK IN GENE LIST | RANK METRIC SCORE | RUNNING ES | CORE ENRICHMENT || 1 | NRP2 |  |  | 9 | 2.539 | 0.0218 | Yes |
| 2 | CLU |  |  | 27 | 2.064 | 0.0377 | Yes |
| 3 | CLSTN1 |  |  | 77 | 1.650 | 0.0435 | Yes |
| 4 | TCF4 |  |  | 96 | 1.551 | 0.0544 | Yes |
| 5 | NEDD4L |  |  | 100 | 1.535 | 0.0680 | Yes |
| 6 | CCDC88A |  |  | 135 | 1.429 | 0.0747 | Yes |
| 7 | MPP6 |  |  | 138 | 1.403 | 0.0873 | Yes |
| 8 | MCF2L |  |  | 194 | 1.272 | 0.0884 | Yes |
| 9 | GOLM1 |  |  | 209 | 1.240 | 0.0972 | Yes |
| 10 | C16orf45 |  |  | 238 | 1.177 | 0.1027 | Yes |
| 11 | KCNQ1OT1 |  |  | 246 | 1.159 | 0.1121 | Yes |
| 12 | PRXL2A |  |  | 259 | 1.130 | 0.1203 | Yes |
| 13 | PFN2 |  |  | 270 | 1.107 | 0.1286 | Yes |
| 14 | PJA2 |  |  | 275 | 1.082 | 0.1379 | Yes |
| 15 | DPYSL2 |  |  | 279 | 1.077 | 0.1473 | Yes |
| 16 | BAZ2B |  |  | 280 | 1.071 | 0.1572 | Yes |
| 17 | FOXO3 |  |  | 291 | 1.054 | 0.1651 | Yes |
| 18 | PCDH9 |  |  | 301 | 1.034 | 0.1729 | Yes |
| 19 | RETREG1 |  |  | 305 | 1.029 | 0.1819 | Yes |
| 20 | BICD1 |  |  | 314 | 1.020 | 0.1898 | Yes |
| 21 | MTURN |  |  | 317 | 1.015 | 0.1989 | Yes |
| 22 | PBX1 |  |  | 332 | 0.987 | 0.2053 | Yes |
| 23 | FOXN3 |  |  | 348 | 0.977 | 0.2115 | Yes |
| 24 | PSD3 |  |  | 350 | 0.975 | 0.2203 | Yes |
| 25 | MSI2 |  |  | 354 | 0.971 | 0.2288 | Yes |
| 26 | NPDC1 |  |  | 375 | 0.932 | 0.2335 | Yes |
| 27 | SESN3 |  |  | 380 | 0.925 | 0.2413 | Yes |
| 28 | ZEB1 |  |  | 385 | 0.920 | 0.2491 | Yes |
| 29 | ZNF91 |  |  | 392 | 0.913 | 0.2564 | Yes |
| 30 | TET1 |  |  | 394 | 0.912 | 0.2647 | Yes |
| 31 | GSE1 |  |  | 418 | 0.885 | 0.2684 | Yes |
| 32 | KDM5B |  |  | 434 | 0.868 | 0.2736 | Yes |
| 33 | DAAM1 |  |  | 444 | 0.860 | 0.2798 | Yes |
| 34 | SMAD1 |  |  | 448 | 0.857 | 0.2872 | Yes |
| 35 | LAPTM4B |  |  | 468 | 0.843 | 0.2913 | Yes |
| 36 | ADCY1 |  |  | 487 | 0.825 | 0.2955 | Yes |
| 37 | APC |  |  | 492 | 0.822 | 0.3023 | Yes |
| 38 | DSEL |  |  | 506 | 0.812 | 0.3073 | Yes |
| 39 | SCRN1 |  |  | 513 | 0.807 | 0.3136 | Yes |
| 40 | ZKSCAN1 |  |  | 524 | 0.794 | 0.3191 | Yes |
| 41 | EFHC1 |  |  | 574 | 0.742 | 0.3164 | Yes |
| 42 | FNBP1L |  |  | 576 | 0.741 | 0.3231 | Yes |
| 43 | TMOD2 |  |  | 591 | 0.732 | 0.3272 | Yes |
| 44 | ENAH |  |  | 601 | 0.726 | 0.3322 | Yes |
| 45 | BRD3 |  |  | 626 | 0.710 | 0.3341 | Yes |
| 46 | SLC39A6 |  |  | 629 | 0.708 | 0.3403 | Yes |
| 47 | BPTF |  |  | 630 | 0.708 | 0.3469 | Yes |
| 48 | ARHGAP21 |  |  | 636 | 0.706 | 0.3524 | Yes |
| 49 | TCEA2 |  |  | 654 | 0.688 | 0.3555 | Yes |
| 50 | ATP6V1B2 |  |  | 674 | 0.674 | 0.3581 | Yes |
| 51 | MUM1 |  |  | 706 | 0.657 | 0.3581 | Yes |
| 52 | PCBP4 |  |  | 719 | 0.649 | 0.3618 | Yes |
| 53 | IP6K2 |  |  | 722 | 0.647 | 0.3674 | Yes |
| 54 | ZNF397 |  |  | 723 | 0.647 | 0.3735 | Yes |
| 55 | ATRX |  |  | 727 | 0.645 | 0.3789 | Yes |
| 56 | PGM2L1 |  |  | 735 | 0.640 | 0.3834 | Yes |
| 57 | CLCN3 |  |  | 759 | 0.624 | 0.3848 | Yes |
| 58 | PPP1R9A |  |  | 768 | 0.621 | 0.3890 | Yes |
| 59 | PNISR |  |  | 769 | 0.621 | 0.3947 | Yes |
| 60 | RALGDS |  |  | 770 | 0.621 | 0.4005 | Yes |
| 61 | KIDINS220 |  |  | 777 | 0.616 | 0.4051 | Yes |
| 62 | TAOK1 |  |  | 786 | 0.609 | 0.4092 | Yes |
| 63 | SRGAP3 |  |  | 789 | 0.609 | 0.4144 | Yes |
| 64 | CASD1 |  |  | 802 | 0.602 | 0.4177 | Yes |
| 65 | EIF4A2 |  |  | 860 | 0.575 | 0.4119 | Yes |
| 66 | PTPRA |  |  | 862 | 0.575 | 0.4171 | Yes |
| 67 | RSBN1L |  |  | 870 | 0.573 | 0.4210 | Yes |
| 68 | HIPK2 |  |  | 896 | 0.563 | 0.4214 | Yes |
| 69 | TULP4 |  |  | 919 | 0.553 | 0.4222 | Yes |
| 70 | CEP170 |  |  | 935 | 0.548 | 0.4244 | Yes |
| 71 | MXI1 |  |  | 944 | 0.546 | 0.4279 | Yes |
| 72 | VAT1 |  |  | 994 | 0.524 | 0.4232 | Yes |
| 73 | CLASP2 |  |  | 1017 | 0.515 | 0.4237 | Yes |
| 74 | GNG2 |  |  | 1019 | 0.515 | 0.4283 | Yes |
| 75 | PRH1 |  |  | 1049 | 0.506 | 0.4273 | Yes |
| 76 | SLC25A36 |  |  | 1061 | 0.503 | 0.4298 | Yes |
| 77 | TMX4 |  |  | 1084 | 0.499 | 0.4302 | Yes |
| 78 | ANKRD12 |  |  | 1132 | 0.485 | 0.4255 | Yes |
| 79 | THRA |  |  | 1138 | 0.484 | 0.4290 | Yes |
| 80 | KIF1B |  |  | 1143 | 0.482 | 0.4327 | Yes |
| 81 | TSPYL1 |  |  | 1150 | 0.480 | 0.4360 | Yes |
| 82 | PRRC2B |  |  | 1152 | 0.479 | 0.4403 | Yes |
| 83 | ZNF292 |  |  | 1153 | 0.479 | 0.4447 | Yes |
| 84 | TANC2 |  |  | 1163 | 0.477 | 0.4474 | Yes |
| 85 | YPEL1 |  |  | 1187 | 0.470 | 0.4473 | Yes |
| 86 | ARGLU1 |  |  | 1191 | 0.469 | 0.4511 | Yes |
| 87 | PARP6 |  |  | 1203 | 0.464 | 0.4532 | Yes |
| 88 | AUTS2 |  |  | 1211 | 0.462 | 0.4561 | Yes |
| 89 | WDR6 |  |  | 1216 | 0.459 | 0.4596 | Yes |
| 90 | CHD9 |  |  | 1220 | 0.458 | 0.4633 | Yes |
| 91 | SLC39A10 |  |  | 1222 | 0.458 | 0.4674 | Yes |
| 92 | DOCK4 |  |  | 1247 | 0.452 | 0.4669 | Yes |
| 93 | FBXO21 |  |  | 1257 | 0.451 | 0.4693 | Yes |
| 94 | MAP1LC3A |  |  | 1313 | 0.438 | 0.4627 | Yes |
| 95 | C5orf24 |  |  | 1315 | 0.437 | 0.4665 | Yes |
| 96 | SECISBP2 |  |  | 1321 | 0.435 | 0.4696 | Yes |
| 97 | KANSL1 |  |  | 1345 | 0.429 | 0.4691 | Yes |
| 98 | ZNF429 |  |  | 1352 | 0.428 | 0.4719 | Yes |
| 99 | ZDHHC21 |  |  | 1371 | 0.423 | 0.4723 | Yes |
| 100 | ORAI2 |  |  | 1389 | 0.419 | 0.4729 | Yes |
| 101 | MAPRE3 |  |  | 1397 | 0.417 | 0.4754 | Yes |
| 102 | CSNK1E |  |  | 1412 | 0.413 | 0.4765 | Yes |
| 103 | MARCH6 |  |  | 1468 | 0.398 | 0.4695 | Yes |
| 104 | MAPK10 |  |  | 1471 | 0.398 | 0.4728 | Yes |
| 105 | DEAF1 |  |  | 1476 | 0.396 | 0.4757 | Yes |
| 106 | DTX3 |  |  | 1479 | 0.395 | 0.4790 | Yes |
| 107 | UBN2 |  |  | 1512 | 0.386 | 0.4763 | Yes |
| 108 | MTMR4 |  |  | 1515 | 0.385 | 0.4795 | Yes |
| 109 | PPP2CB |  |  | 1580 | 0.370 | 0.4705 | No |
| 110 | NUDT3 |  |  | 1581 | 0.370 | 0.4739 | No |
| 111 | PCBD1 |  |  | 1625 | 0.363 | 0.4689 | No |
| 112 | NOL4L |  |  | 1642 | 0.359 | 0.4691 | No |
| 113 | USP11 |  |  | 1665 | 0.356 | 0.4681 | No |
| 114 | FSD1 |  |  | 1701 | 0.349 | 0.4645 | No |
| 115 | GSTA4 |  |  | 1702 | 0.349 | 0.4678 | No |
| 116 | MARCKS |  |  | 1713 | 0.348 | 0.4690 | No |
| 117 | ZC2HC1A |  |  | 1730 | 0.344 | 0.4691 | No |
| 118 | SPAG9 |  |  | 1836 | 0.326 | 0.4517 | No |
| 119 | ATXN7L3B |  |  | 1844 | 0.325 | 0.4533 | No |
| 120 | DBN1 |  |  | 1846 | 0.325 | 0.4561 | No |
| 121 | EXOC4 |  |  | 1852 | 0.323 | 0.4582 | No |
| 122 | TRIM13 |  |  | 1880 | 0.316 | 0.4558 | No |
| 123 | PTPRN2 |  |  | 1902 | 0.314 | 0.4547 | No |
| 124 | PLD3 |  |  | 1913 | 0.312 | 0.4556 | No |
| 125 | ABR |  |  | 1947 | 0.306 | 0.4520 | No |
| 126 | HIST3H2A |  |  | 2034 | 0.292 | 0.4380 | No |
| 127 | TSC2 |  |  | 2042 | 0.290 | 0.4393 | No |
| 128 | CTTNBP2NL |  |  | 2067 | 0.287 | 0.4373 | No |
| 129 | SMARCA1 |  |  | 2078 | 0.286 | 0.4380 | No |
| 130 | FKBP1B |  |  | 2122 | 0.281 | 0.4322 | No |
| 131 | VEZF1 |  |  | 2161 | 0.274 | 0.4273 | No |
| 132 | SLC35G2 |  |  | 2171 | 0.272 | 0.4281 | No |
| 133 | TSPYL4 |  |  | 2189 | 0.269 | 0.4273 | No |
| 134 | CPNE2 |  |  | 2202 | 0.267 | 0.4274 | No |
| 135 | PLEKHA8 |  |  | 2246 | 0.262 | 0.4215 | No |
| 136 | APBB1 |  |  | 2278 | 0.258 | 0.4178 | No |
| 137 | ZNF37A |  |  | 2301 | 0.255 | 0.4159 | No |
| 138 | SPIN1 |  |  | 2358 | -0.254 | 0.4073 | No |
| 139 | GSTM3 |  |  | 2463 | -0.265 | 0.3895 | No |
| 140 | PRKACB |  |  | 2494 | -0.269 | 0.3862 | No |
| 141 | TSHZ2 |  |  | 2517 | -0.272 | 0.3844 | No |
| 142 | KIAA0232 |  |  | 2529 | -0.272 | 0.3848 | No |
| 143 | KIAA0930 |  |  | 2728 | -0.297 | 0.3489 | No |
| 144 | ANK3 |  |  | 3216 | -0.363 | 0.2573 | No |
| 145 | EPHB6 |  |  | 3391 | -0.389 | 0.2270 | No |
| 146 | CDC42EP4 |  |  | 3556 | -0.420 | 0.1989 | No |
| 147 | CEP290 |  |  | 3977 | -0.505 | 0.1217 | No |
| 148 | CNRIP1 |  |  | 4057 | -0.526 | 0.1112 | No |
| 149 | AHI1 |  |  | 4239 | -0.576 | 0.0812 | No |
| 150 | HPCAL1 |  |  | 4378 | -0.627 | 0.0601 | No |
| 151 | TMSB10 |  |  | 4451 | -0.654 | 0.0521 | No |
| 152 | RAP1GAP2 |  |  | 4454 | -0.655 | 0.0578 | No |
| 153 | TLN2 |  |  | 4475 | -0.667 | 0.0601 | No |
| 154 | DNM1 |  |  | 4528 | -0.696 | 0.0565 | No |
| 155 | EFNB2 |  |  | 4610 | -0.738 | 0.0475 | No |
| 156 | UBE2E3 |  |  | 4632 | -0.757 | 0.0505 | No |
| 157 | LSP1P5 |  |  | 4634 | -0.759 | 0.0573 | No |
| 158 | PDE4DIP |  |  | 4715 | -0.804 | 0.0492 | No |
| 159 | LARP6 |  |  | 4785 | -0.849 | 0.0436 | No |
| 160 | PKIB |  |  | 4807 | -0.873 | 0.0476 | No |
| 161 | SHC1 |  |  | 4885 | -0.948 | 0.0414 | No |
| 162 | RAB3B |  |  | 5105 | -1.312 | 0.0109 | No |
| 163 | ALCAM |  |  | 5118 | -1.327 | 0.0209 | No |
| 164 | SORBS2 |  |  | 5124 | -1.342 | 0.0324 | No |
Table: GSEA details [plain text format]

  

Fig 2: NB\_KI\_CLUSTER2: Random ES distribution      
 Gene set null distribution of ES for **NB\_KI\_CLUSTER2**
